# Supplementary material for: Essential and dual effects of Notch activity on a natural transdifferentiation event
Source: Nat Commun. 2025 Jan 2;16:75. doi: 10.1038/s41467-024-55286-8 (PMC11697417; doi:10.1038/s41467-024-55286-8)
Supplement: Supplementary file 1 — Supplementary Information [file 41467_2024_55286_MOESM1_ESM.pdf]

Essential and dual effects of Notch activity on a natural transdifferentiation event

**A**	Genotype	[1PDA]	[0PDA]	[2PDA]	Sterility	n=
loss-of-function	*glp-1(e2141ts)*	100%	0%	0%	100%	386
gain-of-function	*glp-1(ar202ts)*	100%	0%	0%	100%	370

B

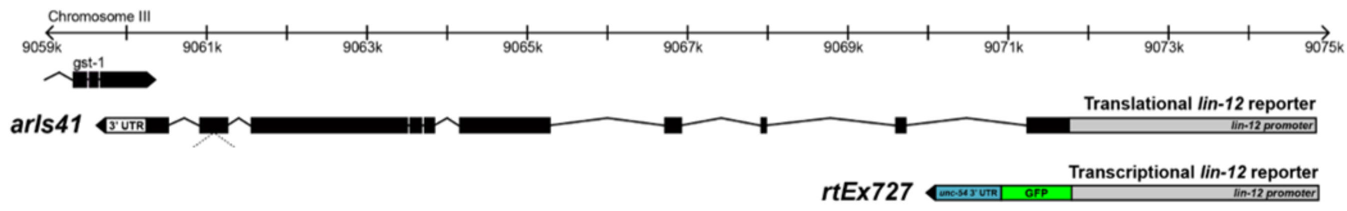

C

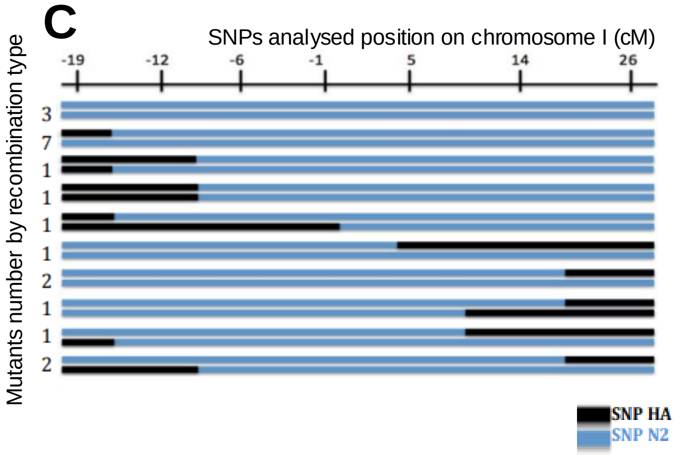

D

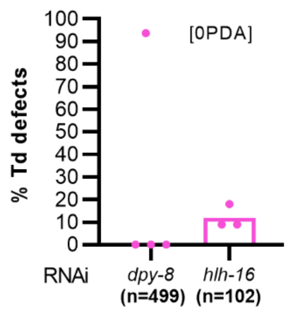

E

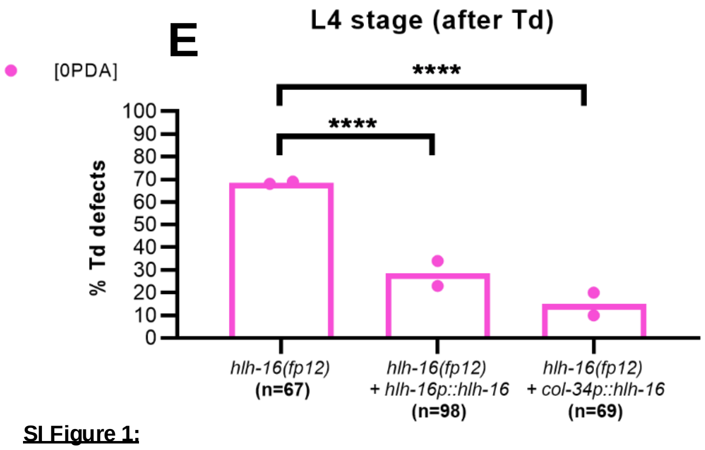

F

L1 stage (before Td)

	col-34		*ceh-6*	
	Y cell present	Y cell absent	Y cell present	Y cell absent
WT	46	0	90	0
hlh-16 (fp12)	155	0	90	0

<u>SI Figure 1:</u>

A) Y-to-PDA defects (in %) in loss and gain-of-function mutants for *glp-1* at the restrictive temperature (assessed using *cog-1::gfp* marker as a PDA marker). Sterility was assessed to verify that worms have been correctly shifted at the restrictive temperature.

B) Reporters used in Fig2A-B. The translational reporter *arIs41* consists of the entire *lin-12* genomic locus (from 3.5kb upstream of the coding region to 0.8 kb downstream) in which GFP has been inserted in frame in the 9th exon; this translational reporter exhibits rescuing activity (Levitan and Greenwald 1998). The transcriptional reporter *rtEx727*, consists of a 3.5kb promoter fragment of the *lin-12* gene driving the expression of GFP followed by *unc-54* 3'UTR (Singh et al. 2011).

C) SNPs cartography of the genomic region containing *fp12* allele on chromosome I. SNPs repartition of every recombinant specific to Hawaiian strain (black) and N2 strain (blue) on chromosome I. The number of recombinant mutants is indicated on the left. *fp12* mutation is located between -1cM and 5cM. This region includes the *hlh-16* gene.

D) RNAi knock-down of *dpy-8* (negative control) and *hlh-16* in the RNAi hypersensitive mutant *rrf-3(pk1426). cog-1::gfp,* PDA marker. The same data are displayed in SI2A.

E) Rescue of [0 PDA] phenotype observed in *hlh-16(fp12)* loss-of-function mutant by over-expression of *hlh-16* genomic region or over-expression of *hlh-16* under the control of *col-34 promoter (*which drives expression in the rectal cells from late embryogenesis (3-fold stage) to Td initiation (Kagias et al. 2012)). *cog-1::gfp,* PDA marker. Two-tailed P value is calculated using a Chi^2 test. ****P < 0.0001, ***P < 0.001, **P < 0.01, *P<0.05.

A, D-E) n, total number of animals scored. Data represent the mean of replicates (graphs: each mean is represented by a dot).

F) Y cell presence at the L1 stage (before the Td initiation – the number of animals scored and displaying 1 or 0 Y cell is indicated) assessed using two Y cell markers: *col-34* and *ceh-6.* In both wild type and *hlh-16(fp12),* the Y rectal cell is always made.

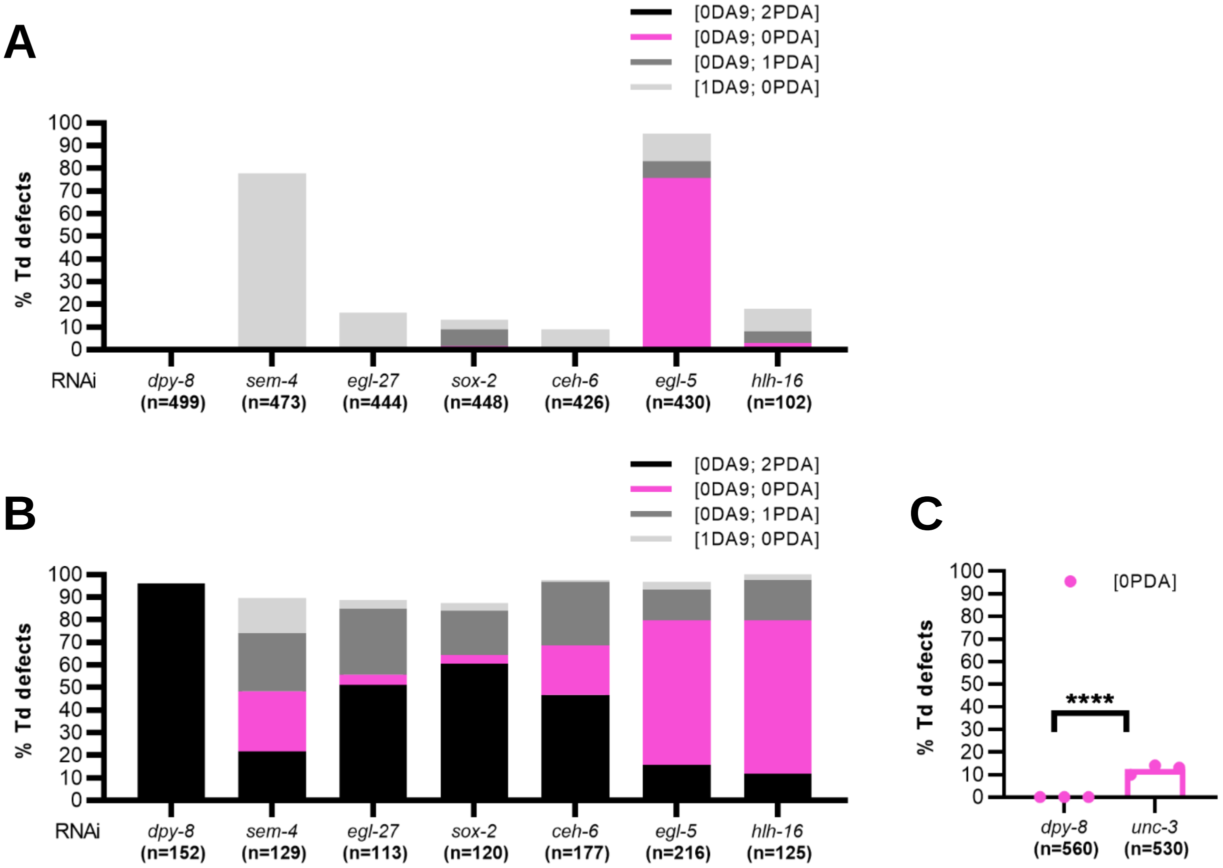

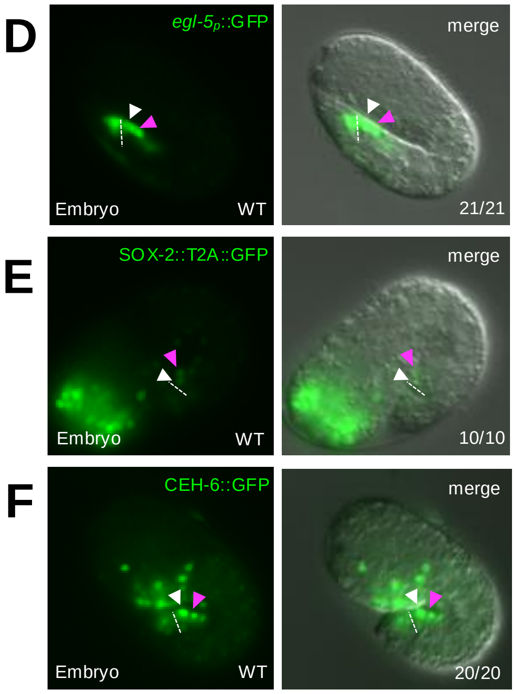

SI figure 2:

A and B) RNAi knock-down of *dpy-8* (negative control), *sem-4, egl-27, ceh-6*, *sox-2, egl-5* and *hlh-16* in (A) the RNAi hypersensitive mutant *rrf-3(pk1426)* and (B) the double mutant *rrf-3(pk1426); lin-12(n950),* carrying a PDA (*cog-1::gfp*) and a DA9 (*itr-1$_p$::mCherry*) markers. n, total number of animals scored.
C) RNAi knock-down of *dpy-8* (negative control) and *unc-3* in the RNAi hypersensitive mutant *rrf-3(pk1426). cog-1::gfp,* PDA marker. n, total number of animals scored.
D) *egl-5$_p$*::GFP is expressed in both Y^{prog} and $DA9^{prog}$ cells in WT embryo (1.7-fold).
E) GFP::T2A::SOX-2 fosmid is expressed in both Y^{prog} and $DA9^{prog}$ cells in WT embryo (1.5-fold).
F) CEH-6::GFP fosmid is expressed in both Y^{prog} and $DA9^{prog}$ cells in WT embryo (1.5-fold).
D-F) White arrowhead: Y cell; magenta arrowhead: DA9. Dotted line: rectal slit. Numbers indicates the fraction of worms displaying this representative expression pattern over the total number of worms scored. Anterior =left. Left panel = fluorescence microscopy; right panel = merged fluorescence and DIC microscopy.
Data in % represent the mean of replicates (the mean of each replicate is represented by a dot). Two-tailed P value is calculated using a Chi^2 test. ****P < 0.0001, ***P < 0.001, **P < 0.01, *P<0.05.

A

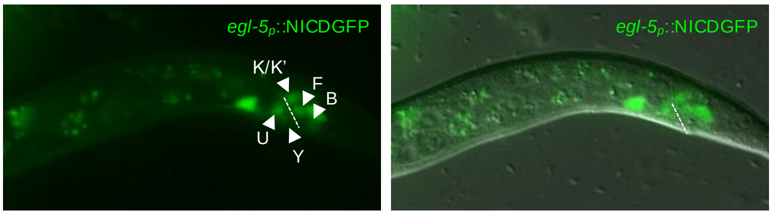

B

Transgene	Nuclear expression/ total animal scored
egl-5$_p$::NICDGFP	45/45
lin-48::NICDGFP	46/46

C

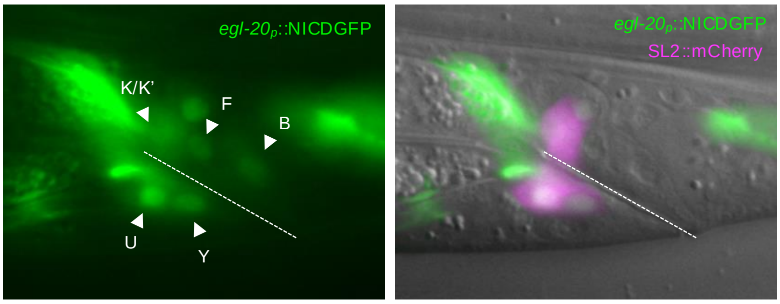

D

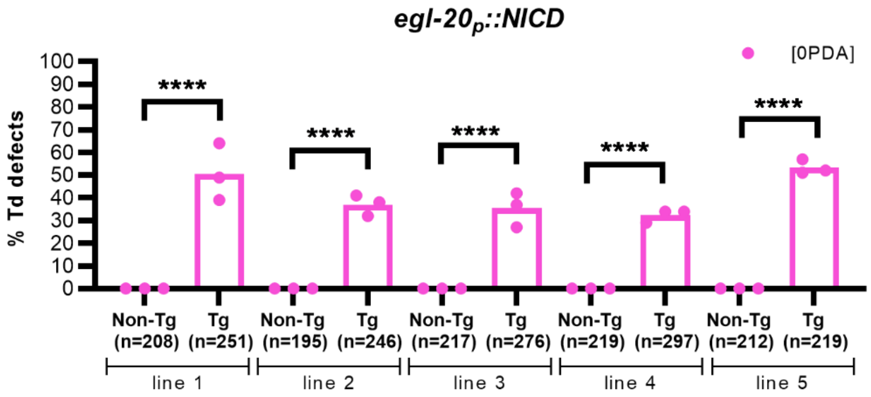

SI Figure 3:

A) NICDGFP, when expressed, is located in the nucleus of the cells (here using *egl-5$_p$::NICDGFP* transgenic L1 worms). Rectal cells are indicated by a white arrowhead and the rectal slit by a dotted line. Anterior = left ; ventral = bottom.

B) Quantification of nuclear NICDGFP expression, scored at the L1 stage in the rectal cells for the indicated transgenes (*fpEx87* and *fpEx337*). Results are expressed as the number of animals showing nuclear expression over the total number of animals showing expression.

C) Wide-spread mosaic expression of *egl-20$_p$::NICDGFP* in all the rectal cells (white arrowheads) including in the Y cell. Representative pattern in a L4 transgenic worm. Dotted line , rectal slit. Anterior = left ; ventral = bottom.

D) Quantification (in %) of [0 PDA] (Td defect) in five independent transgenic lines expressing *egl-20$_p$::NICDGFP::SL2mCherry*.

Tg, transgenic worms; Non-tg, non-transgenic siblings. n, total number of animals scored. Data represent the mean of at least three biological replicates (the mean of each replicate is represented by a dot). Two-tailed P value is calculated using a Chi^2 test. ****$P < 0.0001$, ***$P < 0.001$, **$P < 0.01$, *$P<0.05$

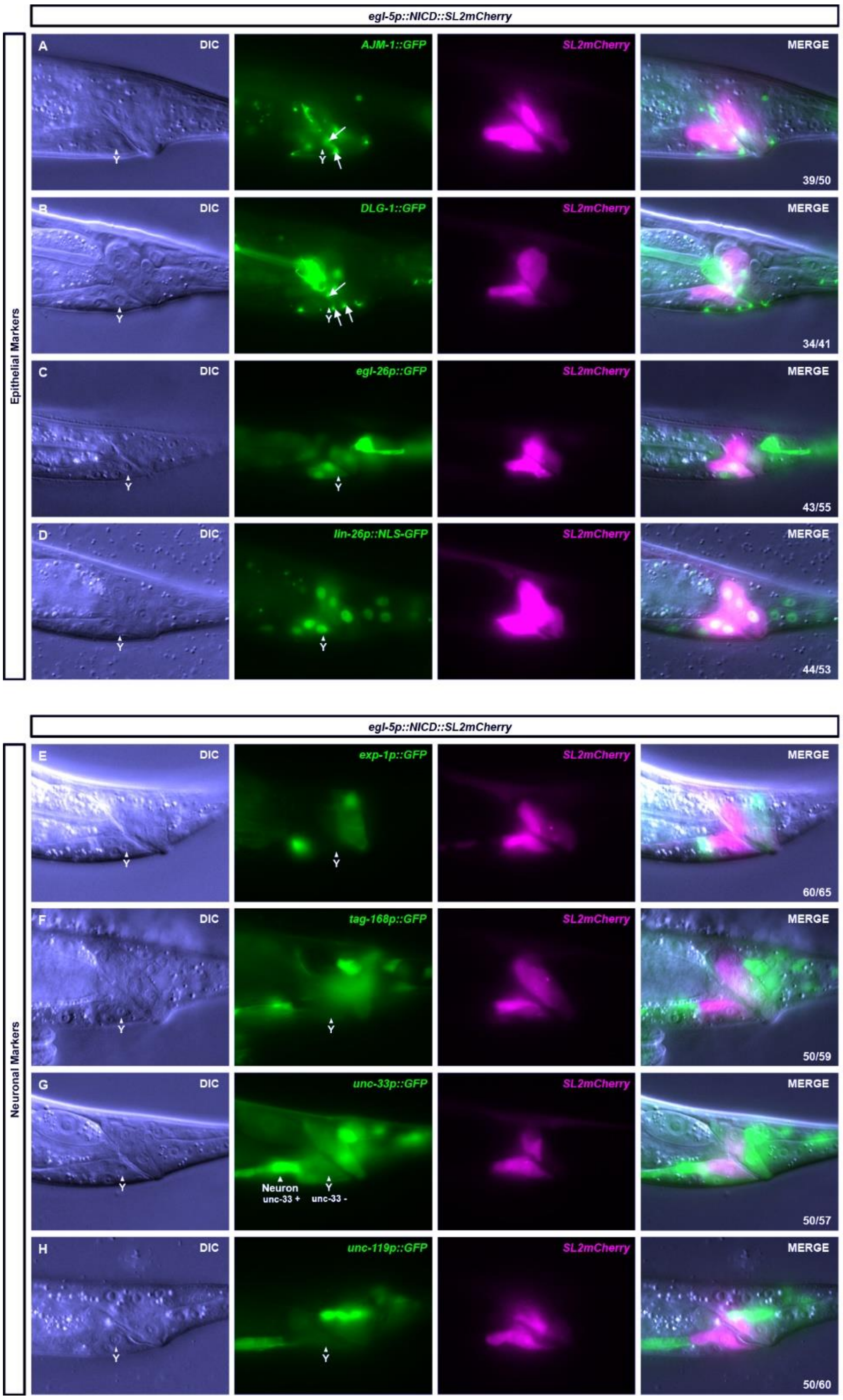

<u>SI Figure 4:</u>

Representative pictures of different epithelial (upper panels) or neuronal (lower panels) markers expression in the persistent Y cell (white arrowhead) when LIN-12^{Notch} signal is maintained throughout (using the *egl-5_p::NICD::SL2::mCherry* transgene). DIC (left), epifluorescence (middle) and merged (right) images in L4 transgenic worms are shown. SL2::mCherry shows NICD expression in the rectal cells. Numbers represent the fraction of worms showing this representative phenotype over the total number of animals scored. Anterior is to the left and ventral to the bottom.

A-B) epithelial junction markers, (A) *AJM-1::GFP* and (B) *DLG- 1::GFP* are expressed (arrows) in the persistent Y (arrowhead).

C) The rectal cell marker, *egl- 26p::GFP*, is expressed in the persistent Y (arrowhead).

D) The epithelial differentiation marker, *lin-26p::NLS-GFP*, is expressed in the persistent Y (arrowhead).

E) The PDA marker, *exp-1_p::GFP*, is not expressed in the persistent Y (arrowhead).

F-G) The pan-neuronal markers (F) *tag-168p::GFP* and (G) *unc-33_p::GFP* are not expressed in the persistent Y (arrowhead). Note that *unc-33_p::GFP* displays a high background around positive cells. Nevertheless, the difference between an *unc-33* positive cell and an *unc-33* negative cell is clear.

H) The pan-neuronal marker *unc-119_p::GFP*, is not expressed in the persistent Y (arrowhead).

A

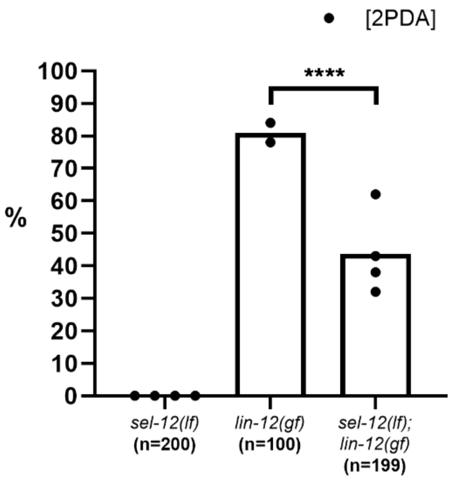

B

	Genotype	RNAi	T°C	PDA OK	No PDA	n =
DSL	***arg-1***(*ok3127)X*	-	25°C	100%	0%	582
	rrf-3(pk1426)II; ***arg-1***(*ok3127)X*	*sel-12*	20°C	100%	0%	582
	dsl-1(*ok810)III*	-	25°C	100%	0%	367
	dsl-1(*ok810)III; sel-12(ar171)unc-1(e538)X*	-	25°C	100%	0%	441
	dsl-3(*ok3411)IV*	-	25°C	100%	0%	389
	dsl-3(*ok3411)IV; sel-12(ar171)unc-1(e538)X*	-	25°C	100%	0%	287
	dsl-4(*ok1020)X*	-	25°C	100%	0%	285
	rrf-3(pk1426); ***dsl-4***(*ok1020)X*	*sel-12*	20°C	100%	0%	697
	dsl-5(*ok588)IV*	-	25°C	100%	0%	349
	dsl-5(*ok588)IV; sel-12(ar171)unc-1(e538)X*	-	25°C	100%	0%	344
	dsl-6(*ok2265)IV*	-	25°C	100%	0%	310
	dsl-6(*ok2265)IV; sel-12(ar171)unc-1(e538)X*	-	25°C	100%	0%	252
DOS	***osm-7***(*n1515)III*	-	25°C	100%	0%	253
	osm-7(*n1515)III; sel-12(ar171)unc-1(e538)X*	-	25°C	100%	0%	308
	osm-11(*rt192)X*	-	25°C	100%	0%	396
	rrf-3(pk1426)II; ***osm-11***(*rt192)X*	*sel-12*	20°C	100%	0%	412
	dos-1(*ok2398)III*	-	25°C	100%	0%	353
	dos-1(*ok2398)III; sel-12(ar171)unc-1(e538)X*	-	25°C	100%	0%	299
	dos-2(*tm4515)II*	-	25°C	100%	0%	480
	dos-2(*tm4515)II; sel-12(ar171)unc-1(e538)X*	-	25°C	100%	0%	306
	dos-3(*tm4899)III*	-	25°C	100%	0%	425
	dos-3(*tm4899)III; sel-12(ar171)unc-1(e538)X*	-	25°C	100%	0%	393

SI Figure 5:

A) Quantification (in %) of [2 PDA] in *sel-12(ar171)* single mutant [noted *sel-12(lf)*] compared to *lin-12(n950)* gain-of-function [noted *lin-12(gf)*] and double *lin-12(n950);sel-12(ar171)* mutants. *sel-12* mutant has a WT PDA while a double mutant *lin-12(n950); sel-12(ar171)* exhibits a reduction of [2 PDA] caused by *lin-12(n950)* and has therefore been hereafter used as a sensitised background for Notch signalling deficiencies. Data represent the mean of biological replicates (the mean of each replicate is represented by a dot). Two-tailed P value is calculated using a Chi^2 test; ****P < 0.0001, ***P < 0.001.

B) Scoring of all the available mutants for non-canonical ligands, alone or associated with *sel-12(ar171)* at the indicated temperature, and expressed in % of total animals scored (n). Data represent biological triplicates.

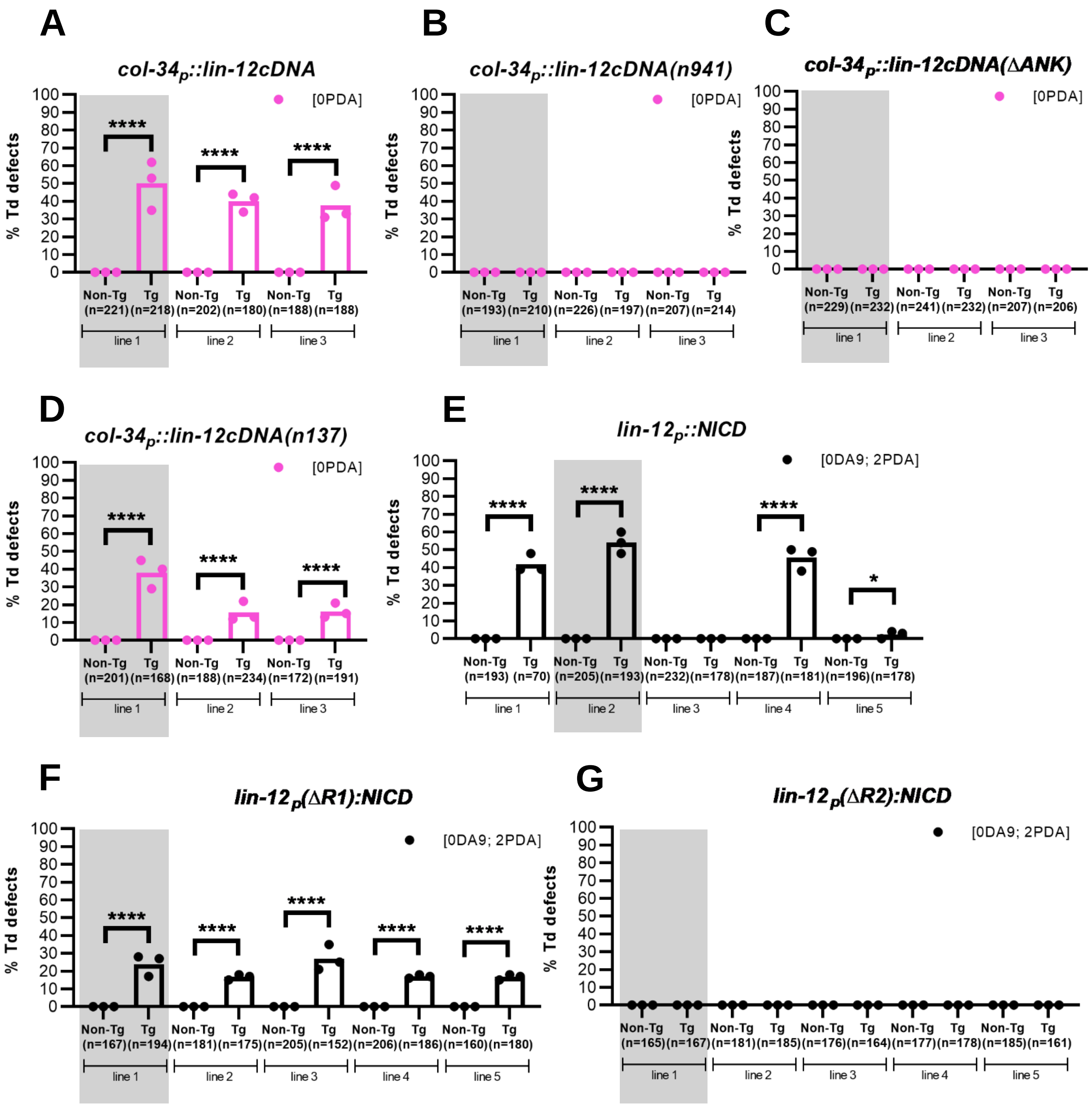

<u>**SI Figure 6:**</u>

A) Quantification of the [0 PDA] phenotype in lines carrying a *col-34*$_p$::*lin-12(WT)*cDNA transgene. When expression of *lin-12* is prolonged until the initiation of Td, Y Td is impaired and a [0 PDA] phenotype appears in transgenic (Tg) worms but not in non-transgenic (non-Tg) control siblings.

B) Quantification of the No Td [0 PDA] phenotype in lines carrying the negative control transgene *col-34*$_p$::*lin-12cDNA(n941).* All the scored worms are phenotypically wild type.

C) Quantification of the [0 PDA] phenotype in lines carrying the negative control transgene *col-34*$_p$::*lin-12cDNA(ΔANK).* All the scored worms are phenotypically wild type.

D) Quantification of the [0 PDA] phenotype in lines carrying the positive control *col-34*$_p$::*lin-12cDNA(n137).* Transgenic worms display a [0 PDA] phenotype.

A-D) Three independent transgenic lines were scored (in %) for each construct. The grey boxes represent the lines depicted in the Figure 6E. For each line, data represent the mean of three biological replicates (each represented by a dot). Tg, transgenic worms. Non-Tg, non-transgenic control siblings. *cog-1::gfp,* PDA marker. n, total number of worms scored. ****P < 0.0001, two-tailed P value using a Chi^2 test.

E) Quantification of the number of PDA and DA9 neurons in lines expressing *lin-12*$_p$::NICD. An additional Td [2 PDA] phenotype appears in transgenics worms in 4 out of 5 lines while all the non-transgenics siblings are WT.

F) Quantification of the number of PDA and DA9 neurons in lines expressing *lin-12*$_p$(ΔR1)::NICD. Deletion of the R1 region does not abolish transgene activity, as a [2 PDA] phenotype can still be obtained.

G) Quantification of the number of PDA and DA9 neurons in lines expressing *lin-12*$_p$(ΔR2)::NICD. Transgene expression appears affected by deletion of the R2 region, as exemplified by the absence of a [2 PDA] phenotype.

E-G) Five independent transgenic lines were scored (in %) for each construct. The grey boxes represent the lines depicted in the Figure 7F. Data represent the mean of replicates (the mean of each replicate is represented by a dot). Tg, transgenic worms; Non-Tg, non-transgenic siblings. *cog-1::gfp,* PDA marker. *itr-1p::mCherry,* DA9 marker. n, total number of worms scored. Two-tailed P value is calculated using a Chi^2 test. ****P < 0.0001, *P < 0.05.

SI Table 1: No « precaucious PDA » is observed in newly hatched L1 with elevated Notch activity, as assessed using *cog-1* expression

Markers	*cog-1* [&]	*hlh-16* [#]	*egl-5* [#]
lin-12(n676n930) 15°C	96/96 no expression	-	-
lin-12(n302)	106/106 no expression	-	-
lin-12(n950)	82/82 no expression	-	-
lin-12p::NICD	100/100 no expression	46/91 (51%) of [2Y]	46/91 (51%) of [2Y]

&, Data expressed as n animal with NO expression / total number of animals scored
[#], Data expressed as n animal with 2 Y cells expressing the marker / total number of animals scored

SI Table 2: *C. elegans* Strains used in this study

Note that the transgenes generated in this study are followed by "*". Refer to SI tables 3 (extrachromosomal arrays) and 4 (integrated arrays) for more details.

Strain Name	Purpose	Reference
Wild-type background		
IS98	*syIs63[cog-1::gfp;unc-119(+)] IV (outcrossed 8x)*	Jarriault *et al.,* 2008
lin-12 mutant characterisation		
IS791	*lin-12(n950) III; syIs63[cog-1::gfp;unc-119(+)] IV*	Jarriault *et al*., 2008
IS164	*lin-12(n302) III; syIs63[cog-1::gfp;unc-119(+)] IV*	This study
IS2120	*syIs63[cog-1::gfp;unc-119(+)] IV; fpEx683[mig-13p::mCherry; myo-2::mCherry]**	This study
IS2121	*syIs63[cog-1::gfp;unc-119(+)] IV; fpEx684[mig-13p::mCherry; myo-2::mCherry]**	This study
IS2123	*syIs63[cog-1::gfp;unc-119(+)] IV; fpEx686[mig-13p::mCherry; myo-2::mCherry]**	This study
IS2118	*lin-12(n950) III; syIs63[cog-1::gfp;unc-119(+)] IV; fpEx682[mig-13p::mCherry; myo-2::mCherry]**	This study
IS2115	*syIs63[cog-1::gfp;unc-119(+)] IV; wyEx1902[itr-1p::mCherry; odr-1p::GFP]*	This study
IS2116	*lin-12(n950) III; syIs63[cog-1::gfp;unc-119(+)] IV; wyEx1902[itr-1p::mCherry; odr-1p::GFP]*	This study
IS2199	*syIs63[cog-1::gfp;unc-119(+)] IV; fpIs67[itr-1p::mCherry; odr-1p::GFP]**	This study
IS2235	*unc-32(e189) lin-12(n676n930)III; syIs63[cog-1::gfp;unc-119(+)] IV; fpIs67[itr-1p::mCherry; odr-1p::GFP]**	This study

glp-1 mutant characterisation		
IS790	*glp-1(e2141ts) III; syIs63[cog-1::gfp;unc-119(+)] IV*	This study
IS2153	*glp-1(ar202ts) IIII; syIs63[cog-1::gfp;unc-119(+)] IV*	This study
hsp::NICDGFP transgenic strains		
IS2093	*syIs63[cog-1::gfp;unc-119(+)] IV; fpEx664[hsp-16.2::NICDGFP; myo-2::mCherry]**	This study
IS2094	*syIs63[cog-1::gfp;unc-119(+)] IV; fpEx665[hsp-16.2::NICDGFP; myo-2::mCherry]**	This study
IS2095	*syIs63[cog-1::gfp;unc-119(+)] IV; fpEx666[hsp-16.2::NICDGFP; myo-2::mCherry]**	This study
RNAi sensitised background		
IS85	*rrf-3(pk1426) II; syIs63[cog-1::gfp;unc-119(+)] IV*	Kagias et al., 2012
IS1676	*rrf-3(pk1426) II ; lin-12(n950) III ; syIs63[cog-1::gfp;unc-119(+)] IV*	This Study
IS2274	*rrf-3(pk1426) II ; fpIs67[odr-1:GFP; itr-1p::mcherry]* ; syIs63[cog-1::gfp;unc-119(+)] IV*	This study
IS2286	*rrf-3(pk1426) II ; lin-12(n950) III ; fpIs67[odr-1:GFP; itr-1p::mcherry]* ; syIs63[cog-1::gfp;unc-119(+)] IV*	This study
hlh-16 mutant characterisation		
IS3	*bxIs7[egl-5::gfp; lin-15(+)] I*	Jarriault *et al.,* 2008
IS3442	*hlh-16(fp12) I; bxIs7[egl-5::gfp; lin-15(+)] I*	This study
IS663	*hlh-16(fp12) I; [cog-1::gfp;unc-119(+)] IV*	This Study
IS2330	*fpEx828[hlh-16p::mcherry::hlh-16::hlh-16 3'UTR(20ng); myo-2::GFP(3ng)] * ; hlh16(fp12) I ; syIs63[cog-1::gfp;unc-119(+)] IV*	This study
IS2332	*fpEx830[col-34p::mcherry::hlh-16::hlh-16 3'UTR(20ng); myo-2::GFP(3ng)] * ; hlh16(fp12) I ; syIs63[cog-1::gfp;unc-119(+)] IV*	This study
IS1299	*gaIs245[col-34p::HIS-24::mCherry; unc-119(+)] ; oxIs12[unc-47::gfp; lin-15(+)] X*	Riva et al., 2022
IS3126	*hlh-16(fp12) I; gaIs245[col-34p::HIS-24::mCherry; unc-119(+)] V; oxIs12[unc-47::gfp; lin-15(+)] X*	This study
IS2677	*hlh-16(fp12) I; fpEx929[ceh-6 locus 13kb (ceh-6 ORF removed= only GFP/transcriptional in strataclone/sens1 -10ng/µl); myo-2::GFP 2ng/µl; pBluescript 200ng/µl]**	This study
IS2592	*fpEx929[ceh-6 locus 13kb [ceh-6 ORF removed= only GFP/transcriptional] in strataclone/sens1 -10ng/µl; myo-2::GFP 2ng/µl; pBluescript 200ng/µl]*	This study
IS318	*hlh-16(fp12) I; syIs63[cog-1::gfp;unc-119(+)] IV*	This study
CB4856	*Hawaiien wild type strain*	Swan et al., 2002
lin-12 reporter strains		

GS4335	*arIs41[LIN-12::gfp; pRF4]*	Levitan *et al.*, 1998
HA2182	*pha-1(e2123)III; rtEx727[lin-12p::gfp, myo-2p::gfp, pha-1(+)]*	Singh *et al.*, 2011
promoter-specific::NICDGFP transgenic strains		
egl-5p::NICDGFP		
IS834	*syIs63[cog-1::gfp;unc-119(+)] IV; fpEx84[egl-5(6,2kb)Δpes10p::NICDGFP::SL2::mCherry, myo-2p::GFP]**	This study
IS835	*syIs63[cog-1::gfp;unc-119(+)] IV; fpEx85[egl-5(6,2kb)Δpes10p::NICDGFP::SL2::mCherry, myo-2p::GFP]**	This study
IS836	*syIs63[cog-1::gfp;unc-119(+)] IV; fpEx86[egl-5(6,2kb)Δpes10p::NICDGFP::SL2::mCherry, myo-2p::GFP]**	This study
IS837	*syIs63[cog-1::gfp;unc-119(+)] IV; fpEx87[egl-5(6,2kb)Δpes10p::NICDGFP::SL2::mCherry, myo-2p::GFP]**	This study
IS838	*syIs63[cog-1::gfp;unc-119(+)] IV; fpEx88[egl-5(6,2kb)Δpes10p::NICDGFP::SL2::mCherry, myo-2p::GFP]**	This study
col-34p::NICDGFP		
IS1052	*syIs63[cog-1::gfp;unc-119(+)] IV; fpEx217[col-34p::NICDGFP; myo-2p::GFP] **	This study
IS1053	*syIs63[cog-1::gfp;unc-119(+)] IV; fpEx218[col-34p::NICDGFP; myo-2p::GFP] **	This study
IS1054	*syIs63[cog-1::gfp;unc-119(+)] IV; fpEx219[col-34p::NICDGFP; myo-2p::GFP] **	This study
IS1055	*syIs63[cog-1::gfp;unc-119(+)] IV; fpEx220[col-34p::NICDGFP; myo-2p::GFP] **	This study
IS1056	*syIs63[cog-1::gfp;unc-119(+)] IV; fpEx221[col-34p::NICDGFP; myo-2p::GFP] **	This study
lin-48p::NICDGFP		
IS1388	*syIs63[cog-1::gfp;unc-119(+)] IV; fpEx334[lin-48p::NICDGFP::SL2::mCherry; myo-2p::GFP]**	This study
IS1389	*syIs63[cog-1::gfp;unc-119(+)] IV; fpEx335[lin-48p::NICDGFP::SL2::mCherry; myo-2p::GFP]**	This study
IS1390	*syIs63[cog-1::gfp;unc-119(+)] IV; fpEx336[lin-48p::NICDGFP::SL2::mCherry; myo-2p::GFP]**	This study
IS1391	*syIs63[cog-1::gfp;unc-119(+)] IV; fpEx337[lin-48p::NICDGFP::SL2::mCherry; myo-2p::GFP]**	This study
IS1392	*syIs63[cog-1::gfp;unc-119(+)] IV; fpEx338[lin-48p::NICDGFP::SL2::mCherry; myo-2p::GFP]**	This study
egl-20p::NICDGFP		

IS1623	*syIs63[cog-1::gfp;unc-119(+)] IV; fpEx459[egl-20p::NICDGFP::SL2::mCherry; myo-2p::mCherry]**	This study
IS1624	*syIs63[cog-1::gfp;unc-119(+)] IV; fpEx460[egl-20p::NICDGFP::SL2::mCherry; myo-2p::mCherry] **	This study
IS1625	*syIs63[cog-1::gfp;unc-119(+)] IV; fpEx461[egl-20p::NICDGFP::SL2::mCherry; myo-2p::mCherry] **	This study
IS1626	*syIs63[cog-1::gfp;unc-119(+)] IV; fpEx462[egl-20p::NICDGFP::SL2::mCherry; myo-2p::mCherry] **	This study
IS1627	*syIs63[cog-1::gfp;unc-119(+)] IV; fpEx463[egl-20p::NICDGFP::SL2::mCherry; myo-2p::mCherry] **	This study
	egl-5p::NICD	
IS1831	*fpIs51[egl-5(6,2kb)Δpes10p::NICD::SL2::mCherry] **	This study
IS1880	*syIs63[cog-1::gfp;unc-119(+)] IV; fpIs51[egl-5(6,2kb)Δpes10p::NICD::SL2::mCherry] **	This study
	Reporter strains used to assess Y identity	
IS1908	*fpIs51[egl-5(6,2kb)Δpes10p::NICD::SL2::mCherry]*; mcIs17[lin-26p::NLS-GFP;pRF4]*	This study
IS1900	*fpIs51[egl-5(6,2kb)Δpes10p::NICD::SL2::mCherry]*; kuIs36[egl-26p::gfp; unc-119(+)]*	This study
IS1904	*jcIs1[AJM-1::GFP; pRF4] IV; fpIs51[egl-5(6,2kb)Δpes10p::NICD::SL2::mCherry] **	This study
IS1902	*fpIs51[egl-5(6,2kb)Δpes10p::NICD::SL2::mCherry] *; mcIs47[DLG-1::gfp; pRF4]*	This study
IS1912	*otIs117[unc-4(+); unc-33p::GFP] IV; fpIs51[egl-5(6,2kb)Δpes10p::NICD::SL2::mCherry]**	This study
IS1911	*ncIs3[tag-168p::GFP] III; fpIs54[egl-5(6,2kb)Δpes10p::NICD::SL2::mCherry] **	This study
IS1910	*edIs6[unc-119p::gfp; rol-6] IV; fpIs51[egl-5(6,2kb)Δpes10p::NICD::SL2::mCherry] **	This study
IS1915	*wyIs75[unc-47p::DsRed; exp-1p::GFP; odr-1p::RFP] III; fpIs51[egl-5(6,2kb)Δpes10p::NICD::SL2::mCherry] **	This study
IS1916	*wyIs75[unc-47p::DsRed; exp-1p::GFP; odr-1p::RFP] III; fpIs54[egl-5(6,2kb)Δpes10p::NICD::SL2::mCherry]**	This study
	Reporter strains used to assess rectal cells identity	
IS1908	*fpIs51[egl-5(6,2kb)Δpes10p::NICD::SL2::mCherry] *; mcIs17[lin-26p::NLS-GFP;pRF4]*	This study
IS2198	*fpIs51[egl-5(6,2kb)Δpes10p::NICD::SL2::mCherry] *; saIs14[lin-48p::GFP; unc-119(+)]*	This study

IS2149	*eIs34[mab-9p::gfp; pCes1943 (rol-6D)] III; fpIs51[egl-5(6,2kb)Δpes10p::NICD::SL2::mCherry] **	This study
Canonical Notch ligand mutant strains		
IS794	*syIs63[cog-1::gfp;unc-119(+)] IV; sel-12(ar171) unc-1(e538) X*	This study
IS792	*lin-12(n950 III; syIs63[cog-1::gfp;unc-119(+)] IV; sel-12(ar171) unc-1(e538) X*	This study
IS1361	*syIs63[cog-1::gfp;unc-119(+)] IV; apx-1(zu347ts)V*	This study
IS1362	*syIs63[cog-1::gfp;unc-119(+)] IV; apx-1(zu347ts)V; sel-12(ar171) unc-1(e538) X*	This study
IS1364	*syIs63[cog-1::gfp;unc-119(+)] IV; lag-2(q420ts)V*	This study
Ligand reporter strains		
GS3795	*dpy-20(e1282) IV; arIs98[apx-1p::2NLS::YFP; ceh-22::gfp; pMH86]*	Li *et al.*, 2010
JK2049	*qIs19[lag-2p::GFP; pRF4] V*	Blelloch *et al.*, 1999
Non-canonical ligand mutant strains		
IS1925	*syIs63[cog-1::gfp;unc-119(+)] IV; arg-1(ok3127)X*	This study
IS2009	*rrf-3(pk1426) II; syIs63[cog-1::gfp;unc-119(+)] IV; arg-1(ok3127)X*	This study
IS770	*dsl-1(ok810) V; fpIs11[exp-1:p:mCherry; myo-2p::GFP]**	This study
IS973	*dsl-1(ok810) IV; sel-12(ar171) unc-1(e538) X; fpIs10[exp-1p::mCherry; myo-2p::GFP]**	This study
IS1895	*wyIs75[unc-47p::DsRed; exp-1p::GFP; odr-1p::RFP] III; dsl-3(ok3411) IV*	This study
IS1894	*wyIs75[unc-47p::DsRed; exp-1p::GFP; odr-1p::RFP] III; dsl-3(ok3411) IV; sel-12(ar171) unc-1(e538) X*	This study
IS1890	*syIs63[cog-1::gfp;unc-119(+)] IV; dsl-4(ok1020)X*	This study
IS1958	*rrf-3(pk1426) II; syIs63[cog-1::gfp;unc-119(+)] IV; dsl-4(ok1020)X*	This study
IS1872	*wyIs75[unc-47p::DsRed; exp-1p::GFP; odr-1p::RFP] III; dsl-5(ok588) IV*	This study
IS1873	*wyIs75[unc-47p::DsRed; exp-1p::GFP; odr-1p::RFP] III; dsl-5(ok588) IV; sel-12(ar171) unc-1(e538) X*	This study
IS1874	*wyIs75[unc-47p::DsRed; exp-1p::GFP; odr-1p::RFP] III; dsl-6(ok2265) IV*	This study
IS1875	*wyIs75[unc-47p::DsRed; exp-1p::GFP; odr-1p::RFP] III; dsl-6(ok2265) IV; sel-12(ar171) unc-1(e538) X*	This study
IS1887	*osm-7(n1515) III; syIs63[cog-1::gfp;unc-119(+)] IV*	This study
IS1888	*osm-7(n1515) III; syIs63[cog-1::gfp;unc-119(+)] IV; sel-12(ar171) unc-1(e538) X*	This study
IS1924	*syIs63[cog-1::gfp;unc-119(+)] IV; osm-11(rt192) X*	This study
IS1960	*rrf-3(pk1426) II; syIs63[cog-1::gfp;unc-119(+)] IV; osm-11(rt192)X*	This study
IS1876	*dos-1(ok2398) III; syIs63[cog-1::gfp;unc-119(+)] IV*	This study

IS1877	*dos-1(ok2398) III; syIs63[cog-1::gfp;unc-119(+)] IV; sel-12(ar171) unc-1(e538) X*	This study
IS1889	*dos-2(tm4515) II; syIs63[cog-1::gfp;unc-119(+)] IV*	This study
IS1878	*dos-2(tm4515) II; syIs63[cog-1::gfp;unc-119(+)] IV; sel-12(ar171) unc-1(e538) X*	This study
IS1879	*dos-3(tm4899) III; syIs63[cog-1::gfp;unc-119(+)] IV*	This study
IS1881	*dos-3(tm4899) III; syIs63[cog-1::gfp;unc-119(+)] IV; sel-12(ar171) unc-1(e538) X*	This study
Transgenic strains used to assess ligand availability over time		
IS2164	*syIs63[cog-1::gfp;unc-119(+)] IV; fpEx713[col-34p::lin-12cDNA(n941); myo-2p::mCherry] **	This study
IS2166	*syIs63[cog-1::gfp;unc-119(+)] IV; fpEx714[col-34p::lin-12cDNA(n941); myo-2p::mCherry] **	This study
IS2169	*syIs63[cog-1::gfp;unc-119(+)] IV; fpEx717[col-34p::lin-12cDNA(n941); myo-2p::mCherry] **	This study
IS2193	*syIs63[cog-1::gfp;unc-119(+)] IV; fpEx729[col-34p::lin-12cDNA(ΔANK); myo-2p::mCherry] **	This study
IS2195	*syIs63[cog-1::gfp;unc-119(+)] IV; fpEx731[col-34p::lin-12cDNA(ΔANK); myo-2p::mCherry] **	This study
IS2196	*syIs63[cog-1::gfp;unc-119(+)] IV; fpEx732[col-34p::lin-12cDNA(ΔANK); myo-2p::mCherry] **	This study
IS2161	*syIs63[cog-1::gfp;unc-119(+)] IV; fpEx710[col-34p::lin-12cDNA(n137); myo-2p::mCherry] **	This study
IS2162	*syIs63[cog-1::gfp;unc-119(+)] IV; fpEx711[col-34p::lin-12cDNA(n137); myo-2p::mCherry] **	This study
IS2163	*syIs63[cog-1::gfp;unc-119(+)] IV; fpEx712[col-34p::lin-12cDNA(n137); myo-2p::mCherry] **	This study
IS2130	*syIs63[cog-1::gfp;unc-119(+)] IV; fpEx689[col-34p::lin-12cDNA; myo-2p::mCherry] **	This study
IS2132	*syIs63[cog-1::gfp;unc-119(+)] IV; fpEx691[col-34p::lin-12cDNA; myo-2p::mCherry] **	This study
IS2133	*syIs63[cog-1::gfp;unc-119(+)] IV; fpEx692[col-34p::lin-12cDNA; myo-2p::mCherry] **	This study
Expression of NODE-like complex members in Y^{prog} and $DA9^{Prog}$		
MH1337	*kuIs34[sem-4::gfp; unc-119(+)]; unc-119(ed3)*	Grant *et al.*, 2000
IS2213	*fpEx476[GFP::ceh-6 fosmid; odr-1::RFP] *; ceh-6(gk665)*	This Study
IS2239	*fpEx756[GFP::sox-2 fosmid; odr-1::rfp]* ; sox-2(ot640) X*	This Study

IS2857	*fpEx974 [NLS :GFP ::T2A ::SEM-4 Fosmid; dsred::coelomocyte]*; fpIs67[itr-1::mCherry; odr-1::GFP]*; syIs63[cog-1::gfp;unc-119(+)] IV*	This Study
IS3	*bxIs7[egl-5::gfp; lin-15(+)]*	Jarriault *et al.,* 2008
IS2540	*fpIs88[hlh-16::GFP; pRF4]**	This Study
IS2807	*fpEx876[lin-12p::ICL::lin-12UTR (0,5ng); myo-2::mCherry (2ng)]* ; fpEx974[NLS::GFP::T2A::sem-4 Fosmid (50ng) ; DsRed::Coelomocyte (50ng)] **	This Study
IS2802	*fpEx876[lin-12p::ICL::lin-12UTR (0,5ng); myo-2::mCherry (2ng)]* ; fpIs100(egl-5(1,3kb)delta pes10::mkate::unc-54 3'UTR; cc:GFP from fpEx970]* ; fpIs88 [hlh-16::GFP translational; pRF4]**	This Study
Extra PDA in *lin-12(gf)* come from a cell that first adopts Y fate		
IS2528	*lin-12(n950) III ; kuIs34[sem-4::gfp; unc-119(+)]*	This Study
Regulation of *lin-12* expression is at the transcriptional level		
IS2443	*fpEx875[lin-12p::NICDGFP::lin-12UTR; myo-2::mCherry] *; syIs63[cog-1::gfp;unc-119(+)] IV ; fpIs67[odr-1:GFP; itr-1p::mcherry]*	This Study
IS2444	*fpEx876[lin-12p::NICDGFP::lin-12UTR; myo-2::mCherry] *; syIs63[cog-1::gfp;unc-119(+)] IV ; fpIs67[odr-1:GFP; itr-1p::mcherry]*	This Study
IS2445	*fpEx877[lin-12p::NICDGFP::lin-12UTR; myo-2::mCherry] *; syIs63[cog-1::gfp;unc-119(+)] IV ; fpIs67[odr-1:GFP; itr-1p::mcherry]*	This Study
IS2446	*fpEx878[lin-12p::NICDGFP::lin-12UTR; myo-2::mCherry] *; syIs63[cog-1::gfp;unc-119(+)] IV ; fpIs67[odr-1:GFP; itr-1p::mcherry]*	This Study
IS2447	*fpEx879[lin-12p::NICDGFP::lin-12UTR; myo-2::mCherry] *; syIs63[cog-1::gfp;unc-119(+)] IV ; fpIs67[odr-1:GFP; itr-1p::mcherry]*	This Study
IS2504	*fpEx891[lin-12p(ΔR1)::NICDGFP::lin-12UTR; myo-2::mCherry] *; syIs63[cog-1::gfp;unc-119(+)] IV ; fpIs67[odr-1:GFP; itr-1p::mcherry]*	This Study
IS2505	*fpEx892[lin-12p(ΔR1)::NICDGFP::lin-12UTR; myo-2::mCherry] *; syIs63[cog-1::gfp;unc-119(+)] IV ; fpIs67[odr-1:GFP; itr-1p::mcherry]*	This Study
IS2506	*fpEx893[lin-12p(ΔR1)::NICDGFP::lin-12UTR; myo-2::mCherry] *; syIs63[cog-1::gfp;unc-119(+)] IV ; fpIs67[odr-1:GFP; itr-1p::mcherry]*	This Study
IS2507	*fpEx894[lin-12p(ΔR1)::NICDGFP::lin-12UTR; myo-2::mCherry] *; syIs63[cog-1::gfp;unc-119(+)] IV ; fpIs67[odr-1:GFP; itr-1p::mcherry]*	This Study
IS2508	*fpEx895[lin-12p(ΔR1)::NICDGFP::lin-12UTR; myo-2::mCherry] *; syIs63[cog-1::gfp;unc-119(+)] IV ; fpIs67[odr-1:GFP; itr-1p::mcherry]*	This Study
IS2509	*fpEx896[lin-12p(ΔR2)::NICDGFP::lin-12UTR; myo-2::mCherry] *; syIs63[cog-1::gfp;unc-119(+)] IV ; fpIs67[odr-1:GFP; itr-1p::mcherry]*	This Study
IS2510	*fpEx897[lin-12p(ΔR2)::NICDGFP::lin-12UTR; myo-2::mCherry] *; syIs63[cog-1::gfp;unc-119(+)] IV ; fpIs67[odr-1:GFP; itr-1p::mcherry]*	This Study

IS2511	*fpEx898[lin-12p(ΔR2)::NICDGFP::lin-12UTR; myo-2::mCherry] *; syIs63[cog-1::gfp;unc-119(+)] IV ; fpIs67[odr-1:GFP; itr-1p::mcherry]*	This Study
IS2512	*fpEx899[lin-12p(ΔR2)::NICDGFP::lin-12UTR; myo-2::mCherry] *; syIs63[cog-1::gfp;unc-119(+)] IV ; fpIs67[odr-1:GFP; itr-1p::mcherry]*	This Study
IS2513	*fpEx900[lin-12p(ΔR2)::NICDGFP::lin-12UTR; myo-2::mCherry] *; syIs63[cog-1::gfp;unc-119(+)] IV ; fpIs67[odr-1:GFP; itr-1p::mcherry]*	This Study
Strains to assess Notch level effect on Y-to-PDA timing		
IS4210	*fpEx686[mig-13p::mCherry (8ng); myo-2::Mcherry (2ng); pBS (190ng)] * ; syIs63[cog-1::gfp;unc-119(+)] IV*	This Study
IS4211	*fpEx686[mig-13p::mCherry (8ng); myo-2::Mcherry (2ng); pBS (190ng)] *; unc-32(e189) III lin-12(n676n930) III ; syIs63[cog-1::gfp;unc-119(+)] IV*	This Study
IS3	*bxIs7[egl-5::gfp; lin-15(+)]*	Jarriault *et al.,* 2008
IS764	*unc-32(e189) III lin-12(n676n930) III ; bxIs7[egl-5::gfp; lin-15(+)] I*	This Study
IS4239	*unc-32(e189) III lin-12(n676n930) III ; hlh-16(syb683[GFP::linker::hlh-16]) I* ; fpEx686[mig-13p::mCherry (8ng); myo-2::Mcherry (2ng); pBS (190ng)] **	This Study
IS4257	*hlh-16(syb683[GFP::linker::hlh-16]) I *; fpEx686[mig-13p::mCherry (8ng); myo-2::Mcherry (2ng); pBS (190ng)] **	This Study
IS4252	*fpEx686[mig-13p::mCherry (8ng); myo-2::Mcherry (2ng); pBS (190ng)] *; ujIs113 [pie-1p::mCherry::H2B::pie-1 3'UTR + nhr-2p::mCherry::his-24::let-858 3'UTR + unc-119(+)] II ; ngn-1(dev137([mNeonGreen::ngn-1]) IV ; unc-32(e189) III lin-12(n676n930) III*	This Study
IS4262	*fpEx686[mig-13p::mCherry (8ng); myo-2::Mcherry (2ng); pBS (190ng)] *; ujIs113 [pie-1p::mCherry::H2B::pie-1 3'UTR + nhr-2p::mCherry::his-24::let-858 3'UTR + unc-119(+)] II ; ngn-1(dev137([mNeonGreen::ngn-1]) IV*	This Study
IS4247	*unc-32(e189) III lin-12(n676n930) III ; muIs62[Pmig-13-mig-13::GFP + lin-15(+)] ; nsIs913[ngn-1p::myr-mKate2 (5 ng/ul); myo-2p::mCherry (2.5 ng/ul); pBlueScript (92.5 ng/ul)] IV (might contain mig-13(mu225) X lin-15(n765ts) X)*	This Study
IS4248	*muIs62[Pmig-13-mig-13::GFP + lin-15(+)] ; nsIs913[ngn-1p::myr-mKate2 (5 ng/ul); myo-2p::mCherry (2.5 ng/ul); pBlueScript (92.5 ng/ul)] IV (might contain mig-13(mu225) X lin-15(n765ts) X)*	This Study
IS563	*lin-12(n302) III ; syIs63[cog-1::gfp;unc-119(+)] IV*	This Study
IS2118	*lin-12(n950) III ; fpEx682[mig-13p::mCherry (8ng); myo-2::Mcherry (2ng); pBS (190ng)]* ; syIs63[cog-1::gfp;unc-119(+)] IV*	This Study
IS2444	*fpEx876[lin-12p::ICL::lin-12UTR (0,5ng); myo-2::mCherry (2ng)] * ; syIs63[cog-1::gfp;unc-119(+)] IV ; fpIs67[odr-1:GFP; itr-1p::mcherry]**	This Study
IS3873	*hlh-16(syb683[GFP::linker::hlh-16]) I* ; fpIs130[pSJ1007-10µg/ml; pBSK+-200ng/ml]N2 ; fpEx876[lin-12p::ICL::lin-12UTR (0,5ng); myo-2::mCherry (2ng)]**	This Study

<u>SI Table 3</u>: Extrachromosomal arrays generated for this study

Transgene	Co-injection marker	Plasmid	Injection in
fpEx683, fpEx684, fpEx686	myo-2::mCherry (2ng/µL)	pCM327 – mig-13p::mCherry::unc-10 3' UTR (8 ng/µL) (a gift from the Shen laboratory)	*syIs63[cog-1::gfp;unc-119(+)] IV*
fpEx682	myo-2::mCherry (2ng/µL)	pCM327 – mig-13p::mCherry::unc-10 3' UTR (8 ng/µL) (a gift from the Shen laboratory)	*lin-12(n950) III; syIs63[cog-1::gfp;unc-119(+)] IV*
fpEx664, fpEx665, fpEx666	myo-2::mCherry (2ng/µL)	pSJ3171 - hsp-16.2::NICDGFP::unc-54 3′UTR (10ng/µL)	*hsf-1(sy441)I*
fpEx84, fpEx85, fpEx86, fpEx87, fpEx88	myo-2p::GFP (5ng/µL)	pSJ6003 - egl-5(6,2kb)Δpes10p::NICDGFP::SL2::mCherry::unc-54 3′UTR (20ng/µL)	*syIs63[cog-1::gfp;unc-119(+)] IV*
fpEx217, fpEx218, fpEx219, fpEx220, fpEX221	myo-2p::GFP (2ng/µL)	pSJ3173 – col-34p::NICDGFP::unc-54 3′UTR (20ng/µL)	*syIs63[cog-1::gfp;unc-119(+)] IV.*
fpEx334, fpEx335, fpEx336, fpEx337, fpEx338	myo-2p::GFP (2ng/µL)	pSJ3169 – lin-48p::NICDGFP::SL2::mCherry::unc-54 3′UTR (20ng/µL)	*syIs63[cog-1::gfp;unc-119(+)] IV.*
fpEx459, fpEx460, fpEx461, fpEx462, fpEx463	myo-2p::mCherry (2ng/µL)	pSJ3162 – egl-20p::NICDGFP::SL2::mCherry::unc-54 3′UTR (20ng/µL)	*syIs63[cog-1::gfp;unc-119(+)] IV*
fpEx713, fpEx714, fpEx717	myo-2p::mCherry (2ng/µL)	pSJ3222 - col-34p::lin-12cDNA(n941)::unc-54 3′UTR (10ng/µL)	*syIs63[cog-1::gfp;unc-119(+)] IV*
fpEx729, fpEx73, fpEx732	myo-2p::mCherry (2ng/µL)	pSJ3223 - col-34p::lin-12cDNA(ΔANK)::unc-54 3′UTR (10ng/µL)	*syIs63[cog-1::gfp;unc-119(+)] IV*
fpEx710, fpEx711, fpEx712	myo-2p::mCherry (2ng/µL)	pSJ3218 - col-34p::lin-12cDNA(n137)::unc-54 3′UTR (10ng/µL)	*syIs63[cog-1::gfp;unc-119(+)] IV*
fpEx689, fpEx691, fpEx692	col-34p::lin-myo-2p::mCherry (2ng/µL)	pSJ3215 - col-34p::lin-12cDNA::unc-54 3′UTR (10ng/µL)	*syIs63[cog-1::gfp;unc-119(+)] IV*
fpEx875, fpEx876, fpEx877, fpEx878, fpEx879	myo-2p::mCherry (2ng/µL)	pSJ3212 – lin-12p::NICDGFP::lin-12UTR (0.5ng/µL)	*syIs63[cog-1::gfp;unc-119(+)] IV; fpIs67[itr-1p::mCherry; odr-1p::GFP]*
fpEx891, fpEx892, fpEx893, fpEx894, fpEx895	myo-2p::mCherry (2ng/µL)	pSJ3240 – lin-12p(ΔR1)::NICDGFP::lin-12UTR (0.5ng/µL)	*syIs63[cog-1::gfp;unc-119(+)] IV; fpIs67[itr-1p::mCherry; odr-1p::GFP]*
fpEx896, fpEx897, fpEx898, fpEx899, fpEx900	myo-2p::mCherry (2ng/µL)	pSJ3242 – lin-12p(ΔR2)::NICDGFP::lin-12UTR (0.5ng/µL)	*syIs63[cog-1::gfp;unc-119(+)] IV; fpIs67[itr-1p::mCherry; odr-1p::GFP]*

fpEx476	myo-2p::mCherry (2ng/µL)	Fosmid 9347172996193398_D03 = ceh-6::GFP::3xFLAG (20ng/µL) (a gift from Mihail Sarov)	*ceh-6(ok3388) I / hT2[qIs48] strain*
fpEx756	odr-1::rfp (50ng/µL)	Fosmid WRM0626aE02 = GFP::sox-2 (10ng/µL)	*sox-2(ot640) X*
fpEx828	myo-2::GFP (3ng/µL)	pSJ821 - hlh-16p::mcherry::hlh-16::hlh-16 3'UTR (20ng/µL)	*hlh16(fp12) I ; syIs63[cog-1::gfp;unc-119(+)] IV*
fpEx830	myo-2::GFP (3ng)	pSJ823 - col-34p::mcherry::hlh-16::hlh-16 3'UTR (20ng/µL)	*hlh16(fp12) I ; syIs63[cog-1::gfp;unc-119(+)] IV*
fpEx929	myo-2::GFP (2ng/µl)	pSJ6334 – 5.6kb ceh-6p::GFP::3.6kb ceh-6 3'UTR (10ng/µl)	N2
fpEx974	dsred::coelomocyte (50ng/µL)	Fosmid WRM0638dE10, modified as NLS::GFP ::T2A::SEM-4 (50ng/µL)	N2

SI Table 4: Integrated arrays generated for this study

Integrated array	Extrachromosomal array	Plasmid name & gene	genetic background
fpIs130	*fpEx1251*	pSJ1007 = egl-5p(6 kb)::2xNLS::mCherry::unc-54 3'utr (10ng/µL)	N2
fpIs88	*otEx4503* from Bertrand et al. 2011	[hlh-16::GFP; pRF4]	N2
fpIs100	*fpEx970*	pSJ834 = egl-5(1,3kb)Δpes10::mkate::unc-54 3'UTR(20ng/µL); cc::GFP (50ng/µL)	N2
fpIs10; fpIs11	*fpEx30*	pSJ503 = exp-1p::mCherry(10ng/µL); myo-2p::GFP (10ng/µL)	N2
fpIs51, fpIs54	*fpEx497*	pSJ6003 = egl-5(6,2kb)Δpes10p::NICD::SL2::mCherry (20ng/µL)	N2
fpIs67	*wyEx1902* from Teichmann & Shen, 2011	[itr-1pB::mCherry; odr-1p::GFP]	N2

SI Table 5: Oligonucleotides used

Oligo name	Sequence	Use
BDM314	AACGGTACCAGAAAAAATGGTTGTTCTGATGTTAGGAGCATTACC	pSJ3177 and pSJ6003 cloning
BDM315	TTGGTACCTCAAAAATAATGAGCTGGTTCGGAGTATCG	pSJ3177 and pSJ6003 cloning
BDN519	ACAGCATGCGACATGTAAAGTACATCCGTTACATC	pSJ3173 cloning
BDN521	CCCCCCGGGTGTATGCAGTGGTGGTTTGG	pSJ3173 cloning
BDT599	ACATGCATGCGGATCCAAAAAACCTGCATTTTTTTCAG	pSJ3169 cloning (*lin-48* promoter)
BDT600	CCCCCCGGGCTGAAATTGAGCAGAGCTGAAAATTTTTG	pSJ3169 cloning (*lin-48* promoter)
Ceh-6pF	ATAAGAATgcggccgcCGTGTTGCTTTAGCACTTCTCCATCCCTTC	pSJ6334 (*ceh-6*

		transcriptional reporter)
Ceh-6pR	ATAGTTTAgcggccgcCAGTTGGGAAGTCCAGGAGCAACGGGGTG	pSJ6334 (*ceh-6* transcriptional reporter)
Ceh-6UTRf	TTTTTTGTGATGCGTATTGATGTAGC	pSJ6334 (*ceh-6* transcriptional reporter)
Ceh-6UTRr	GTCGACACAGAAACTACGCAAAATC	pSJ6334 (*ceh-6* transcriptional reporter)
mcm239F	TAGAGGATCCccgGGGATTGGCC	pSJ834 cloning (mkate insertion)
mcm239R	GATATTATACATATTTCATAAAGCCAACC	pSJ834 cloning (mkate insertion)
oCG521	CGAGCTCAGAAAAAATGACAGCACCGAAAAAAAGCGAAAAGTTCCAGCTGAGAAGATGACCGCTCCAAAGAAGAAACGCAAAGTA	pSJ1007 cloning
oCG522	CCGGTACTTTGCGTTTCTTCTTTGGAGCGGTCATCTTCTCAGCTGGAACTTTTCGCTTTTTTTCGGTGCTGTCATTTTTTCTGAGCTCGGTAC	pSJ1007 cloning
TD004	GCGGCCGCGCTGTCTCATCCTACTTTCACC	pSJ3169 cloning (*SL2::mCherry*)
TD005	GCGGCCGCCTACTTATACAATTCATCCATGCC	pSJ3169 cloning (*SL2::mCherry*)
TD061	AAAGCATGCGAAGTCATCCTACTAACTAACAATATGACGC	pSJ3162 cloning (*egl-20* promoter)
TD062	AAACCCGGGTATTTCTGAAATTGAGATGTTTTAGAATTTC	pSJ3162 cloning (*egl-20* promoter)
TD065	GACTCAACTCATCTGACACCTCC	pSJ3103 cloning
TD066	GGGTCGAGTTACTTTTCTTGAAGG	pSJ3103 cloning
TD088	taatacgactcactatagggATGCCTTCCACAAGGAGACAAC	*sel-12* gDNA PCR amplification for RNAi experiments
TD089	taatacgactcactatagggGAGATCGCTCAAGATATAATCGAAAAG	*sel-12* gDNA PCR amplification for RNAi experiments
TD094	AAAAGGTACCATGCGGATCCCTACGATTTG	pSJ3215 cloning
TD095	TTTTGGTACCTCAAAAATAATGAGCTGGTTCGG	pSJ3215 cloning
TD101	GTGTTGTTGACTCAATATTTGCAAGGCTTGC	pSJ3218 cloning

TD102	GCAAGCCTTGCAAATATTGAGTCAACAACAC	pSJ3218 cloning
TD105	GGATTCGGTGGGAAATAGTGTGACGAGCCATTG	pSJ3222 cloning
TD106	CAATGGCTCGTCACACTATTTCCCACCGAATCC	pSJ3222 cloning
TD113	CCAGAACGAGAATATTCAATGGATC	pSJ3223 cloning
TD114	AGGTTCAGGTTCAGTTGGAATTTG	pSJ3223 cloning
TD096	CTCAACAGACTTTGCTCAATTTCAAAAAATGGTTGTTCTGATGTTAG GAGCATTAC	pSJ3212 cloning
TD097	GGAATTTAAATAATAAATGACGATTGTTCAGAAGATGTACCGAGCT CGGATCCACTAGTAAC	pSJ3212 cloning
TD136	ACAGTAACAGACACCTGTGCTCC	pSJ3240 cloning
TD137	TATTGTTAATAAATGAGTGTAACATTTAAG	pSJ3240 cloning
TD138	ATTAATGATAATGCAAAAGCTACCAGG	pSJ3242 cloning
TD139	TGTGTCAGTTTTAGAGTTTTATTTCTG	pSJ3242 cloning
